# Vitamin D_3_ Modulates Impaired Crosstalk Between RANK and Glucocorticoid Receptor Signaling in Bone Marrow Cells After Chronic Prednisolone Administration

**DOI:** 10.3389/fendo.2018.00303

**Published:** 2018-06-07

**Authors:** Ihor Shymanskyi, Olha Lisakovska, Anna Mazanova, Dmytro Labudzynskyi, Mykola Veliky

**Affiliations:** Department of Biochemistry of Vitamins and Coenzymes, Palladin Institute of Biochemistry, National Academy of Sciences of Ukraine, Kyiv, Ukraine

**Keywords:** prednisolone, osteoporosis, vitamin D, vitamin D receptor, glucocorticoid receptor, RANKL/RANK/osteoprotegerin axis, nuclear factor kappa-B, osteoclastogenesis

## Abstract

The effectiveness of vitamin D_3_ (cholecalciferol) in counteracting the side effects of glucocorticoid (GC) therapy has been demonstrated previously. Abnormalities in systemic hormonal and local (cytokine) regulation of bone marrow (BM) cells may underlie GC-induced imbalance between osteosynthesis and bone resorption. The cytokine system receptor activator of nuclear factor kappa-B (RANK)/RANK ligand (RANKL)/osteoprotegerin (OPG) is considered as an integrating link in the NF-κB-mediated interaction of various cells involved in maintaining osteoblastic-osteoclastic balance, which makes it a pharmacological target for regulation and correction of the bone remodeling process. We studied GC-induced impairments of the RANKL/RANK/OPG axis in BM cells depending on vitamin D bioavailability and whether these changes were mediated by glucocorticoid (GR) and/or vitamin D (VDR) receptors. Female Wistar rats administered with prednisolone (5 mg/kg b.w., 30 days) showed a decrease in the GR protein level and the number of GR-positive BM cells. GC caused a marked elevation of RANKL and RANK levels in BM, while OPG decreased. Flow cytometry data indicated GC-elicited increase in the number of circulating RANK-positive osteoclast precursors (OCPs) in BM, peripheral blood, and spleen. In full accordance with the data that the interaction of RANKL-RANK leads to transcriptional activation of NF-κB and subsequent differentiation of osteoclasts, we found an increase in the level of phosphorylated p65 subunit of NF-κB with a simultaneous decrease in the NF-κB inhibitor (IκB) level. These changes were accompanied by vitamin D insufficiency and downregulated expression of CYP27B1 and VDR, which are responsible for synthesis and hormonal signaling of 1,25(OH)_2_D. Notably, we observed VDR and RANK co-localization in OCPs. Cholecalciferol co-administration (1,000 IU/kg b.w., 30 days) with prednisolone resulted in elevated GR synthesis in BM. Cholecalciferol prevented prednisolone-elicited disturbances of the RANKL/RANK/OPG, which correlated with improved bioavailability and vitamin D signaling through VDR. This caused the lowering of phosphoNF-κB p65 level and inhibiting NF-κB translocation to the nucleus that could reduce the circulating OCPs pool in BM, peripheral blood, and spleen. Our findings suggest that prednisolone-induced abnormalities in GR and RANKL/RANK/OPG signaling pathways are associated with the impairments of vitamin D auto/paracrine system in BM cells and can be ameliorated by cholecalciferol supplementation.

## Introduction

Glucocorticoids (GCs) have been extensively used in clinical applications as an effective therapy for a variety of severe inflammatory and autoimmune disorders (1, 2). However, long-term or high dose administration of GCs causes osteoporosis, which is characterized by a rapid and severe bone loss and microarchitectural changes in bone tissue, resulting in easy fracturing, and even disability (3). Deleterious side effects of GCs on multi-system pathways linked to osteoblast and osteoclast differentiation and apoptosis, marrow adipogenesis, mineral, and lipid metabolism are the most prevalent pathological features of glucocorticoid-induced skeletal disorders (4).

Several studies suggested that pathogenic mechanisms of GC-associated osteoporosis involve an initial acceleration of osteoclast-induced bone resorption followed by a decrease in osteoblast-mediated bone formation and attenuation of osteoclasts activity. Nevertheless, the exact mechanisms are still controversial and not conclusive. Although GCs are known to affect osteoblast differentiation and function, there have been conflicting reports about the effect of GCs on osteoclast formation and activity, leading to the assumption that the character of their influence depends largely on which synthetic GC is applied, its dosage and treatment duration. It was demonstrated that the differentiation deficiencies of osteoclast precursors (OCPs) may contribute to corticosteroid osteoporosis; however, the stimulating effect of GCs on these cells have also been reported. The latter was limited to the early phase of osteoclast differentiation and the enhanced priming of osteoclast progenitors [bone marrow (BM)-derived monocytes/macrophages] toward differentiation into mature osteoclasts (5).

Bone disruption associated with GC action may be attributed to abnormal regulation of bone remodeling at systemic (hormonal) and local (cytokine) levels. In the complex system of bone remodeling, the receptor activator of nuclear factor κB ligand (RANKL)/osteoprotegerin (OPG) pathway is the coupling factor between bone formation and bone resorption. RANKL, one of the tumor necrosis factor (TNF) superfamily members, is a potent stimulator of osteoclast formation and bone resorption, which acts through the RANK (receptor activator of nuclear factor κB—NF-κB) and often contributes to the pathologies of bone metabolism. RANKL belongs to the group of regulatory glycoproteins that is produced by BM stromal cells, osteoblasts, activated dendritic cells, and T-lymphocytes (6). This emphasizes the importance of RANKL-dependent and NF-κB-mediated osteoimmune interaction in the process of bone tissue remodeling. OPG, synthesized by various cells, acts as a decoy receptor for the RANKL and prevents its osteoclastogenic activity. The proper balance between RANKL and OPG determines the degree of proliferation and osteoclastic activity (7) and impaired OPG to RANKL ratio can be considered as an essential pathogenetic factor in the development of osteoporosis.

Available scientific data concerning the effects of GCs on osteoprogenitors or mature bone tissue cells are mainly obtained in studies on cell cultures or related to changes in bone tissue associated predominantly with short-term administration of high doses of synthetic hormones. Some studies have shown that GCs inhibit the expression of the OPG mRNA and further protein synthesis and secretion, thus leading to the RANKL mRNA overexpression in the culture of osteoblastic cells regardless of the differentiation stage (8). Because glucocorticoid response elements were found within the RANKL gene, the expression of this cytokine may in all probability be regulated by enhanced transcriptional activity of the corticosteroid (9). GCs were also shown to promote osteoclastogenesis by transrepressing the OPG gene through the AP-1 site (10). Although the components of RANKL/RANK signaling pathway are synthesized by the precursor cells of osteoblasts and osteoclasts in the BM; however, it remains an open question whether changes in this cytokine system may lead to the defective osteosynthesis in GC-induced osteoporosis.

Vitamin D_3_ (cholecalciferol) is a unique compound, which is effectively used in the treatment of bone diseases. As a prohormone, it fulfills its biological effects through the mechanism of hormonal action, and as such it cannot be considered a classic vitamin. The hormonally active form of vitamin D_3_ is 1,25-dihydroxyvitamin D_3_ (1,25(OH)_2_D_3_), which through its specific receptor (VDR) provides transcriptional regulation of the expression of about 500 genes in human cells (11). Less recognized is that vitamin D_3_, like other nuclear steroids, can also exert non-genomic effects in various tissues (12).

VDR is a zinc-finger containing protein of the nuclear hormone receptor superfamily. Mechanistically, ligand binding induces conformational changes in VDR that promote receptor heterodimerization with the retinoid X receptor (RXR). After ligand activation, it binds directly to the vitamin D response elements and recruits a variety of co-regulatory complexes that perform the additional functions to modify transcriptional activity (13). Importantly, VDR-mediated gene regulation requires the involvement of multiple modular enhancers at a range of locations many kilobases upstream, downstream, or within the transcription units (14).

One of the main biological functions of vitamin D_3_ is to ensure the normal growth and development of bones as well as the prevention of rickets and osteoporosis. Cholecalciferol regulates *in vivo* mineral metabolism and promotes the deposition of calcium in bone tissue. This action of vitamin D_3_ is provided by its hormonal effect on calcium homeostasis and VDR-mediated regulation of proliferation, differentiation, and apoptosis of various cell types involved in osteogenesis (osteoblasts, osteoclasts, osteocytes, immunocompetent cells). Nevertheless, the molecular mechanisms by which 1,25(OH)_2_D_3_ stimulates bone resorption were also discovered. It has been demonstrate that regulation of *Rankl* gene expression by 1,25(OH)_2_D_3_ is mediated by at least five distal regions in osteoblastic cells that, in addition to the GC receptor, contain binding sites for VDR and RXR (15). *In vitro* exposure of osteoblastic cells to 1,25(OH)_2_D_3_ stimulates RANKL expression, which in turn induces osteoclastogenesis (16). Other results suggest that 1,25(OH)_2_D_3_ can increase bone resorption by directly enhancing the formation and maturation of osteoclasts (17). Thus, recent advances in bone cells and vitamin D_3_ biology have led to a more detailed understanding of bone tissue formation/resorption pathways and clear difference between *in vitro* (osteoclastogenic) and *in vivo* (antiresorptive) effects of active vitamin D_3_ metabolites have been demonstrated.

The urgent scientific problem is to elucidate the role of VDR-mediated signaling in the impairment of osteoblastic–osteoclastic interaction, which provides the realization of bone tissue remodeling and maintenance of bone homeostasis in various pathologies of bone tissue, including GC-induced osteoporosis. Despite the decisive role of vitamin D_3_ and its receptor in the process of bone remodeling, it remains controversial whether the interaction of vitamin D_3_ with the signaling pathways of glucocorticoid receptor (GR) and RANKL/RANK/OPG has any effect on the differentiation of the OCPs after the concurrent administration of cholecalciferol and GCs. In this study, we examined the role of vitamin D_3_ in the regulation of RANKL/RANK/OPG axis in primary BM cells and its possible relationship with abnormal interaction between GR and VDR signaling pathways in the BM after chronic administration of synthetic GC prednisolone.

## Materials and Methods

### Experimental Design

A total of 45 four-week-old female Wistar rats (100 ± 5 g) were randomly divided into the following groups: (1) the control group; (2) the prednisolone group that received orally synthetic GC prednisolone at dose 5 mg/kg of b.w. for 30 days; and (3) the group that received concurrently prednisolone (5 mg/kg of b.w.) and vitamin D_3_ (1,000 IU/kg of b.w. for 30 days, orally). All experiments were conducted in accordance with the international recommendations of the European Convention for the Protection of Vertebrate Animals used for Research and Scientific Purposes (Strasbourg, 1986) and are ethically acceptable. The protocol of animal experiments was approved by the ethics committee on controlling the rules of research work with experimental animals of the Palladin Institute of Biochemistry, Kyiv, Ukraine.

### Total, Nuclear, and Cytoplasmic Protein Extract Preparation and Western Blot Analysis

Total protein extracts were prepared from frozen BM samples using standard protocol with RIPA buffer (20 mM Tris–HCl, pH 7.5; 150 mM NaCl; 1% Triton X-100; 1 mM EGTA; 0.1% SDS, 1% sodium deoxycholate, 10 mM sodium pyrophosphate). Briefly, BM samples (100 mg) were lysed for 20 min in RIPA buffer in the presence of protease inhibitor cocktails (PIC, Sigma, USA), then centrifuged for 20 min (14,000 *g*) at +4°C. Nuclear and cytoplasmic protein fractions were isolated by the method as described (18). Frozen BM samples (100 mg) were ground in a porcelain mortar with liquid nitrogen and lysed by incubation on ice for 20 min in 0.9 ml of 0.5% NP-40-phosphate-buffered saline (PBS) containing PIC. The cell lysates were centrifuged at 1,500 *g* for 5 min, and cytoplasmic proteins were separated from nuclei and further centrifuged at 14,000 *g* for 20 min at +4°C. The nuclei pellets were washed with 0.5% NP-40-PBS containing the PIC and centrifuged at 1,500 *g* for 5 min twice, then mixed with RIPA buffer and PIC by vortexing at +4°C, followed by centrifugation at 14,000 *g* for 20 min. Protein concentration of all supernatants were determined by Lowry’s method. Total, cytoplasmic, and nuclear lysates were stored at −80°C. Equal amounts of protein (for total lysates—50 µg, nuclear fraction—40 µg, cytoplasmic fraction—50 µg) were loaded and separated by 10–15% SDS polyacrylamide gels (depending on the molecular weight of target proteins), followed by transfer of proteins onto nitrocellulose membranes. Membranes were blocked with 5% non-fat milk in PBS plus 0.05% Tween-20 (PBST) followed by incubation overnight with primary antibodies against RANK (1:400; Santa Cruz Biotechnology, USA), RANKL (1:250; Santa Cruz Biotechnology, USA), OPG (1:250; Santa Cruz Biotechnology, USA), CYP27B1 (1:200; Santa Cruz Biotechnology, USA), NF-κB p65 (1:250; Thermo Fisher Scientific Inc., USA), NF-κB p65 phosphorylated at Ser 311 (1:200; Santa Cruz Biotechnology, USA), IκB (1:500; Santa Cruz Biotechnology, USA), GR (1:250; Santa Cruz Biotechnology, USA), β-actin and lamin B1 (1:20,000 and 1:1,000, respectively; Sigma, USA) in PBS supplemented with 0.1% (vol/vol*)* Tween-20 and 5% (wt/vol) non-fat milk. Primary-antibody-bound membranes were then incubated with peroxidase-conjugated secondary antibodies: anti-mouse IgG (Fab Specific)-Peroxidase (1:2,500; Sigma, USA), anti-rabbit IgG (H + L)-HRP conjugate (1:4,000; Bio-Rad Laboratories, Inc., USA) or anti-goat IgG (H + L) (1:2,500; Invitrogen, USA). Thereafter the membranes were developed with chemiluminescent agents: p-coumaric acid (Sigma, USA) and luminol (AppliChem GmbH, Germany). Tissue levels of target proteins in total and cytoplasmic lysates were normalized to β-actin; the levels of target proteins in nuclear lysates were normalized to lamin B1. The immunoreactive bands were quantified with Gel-Pro Analyzer32, v3.1.

### RNA Isolation and Real-Time Quantitative Polymerase Chain Reaction (RT-qPCR)

Total RNAs from BM were obtained using the innuPREP RNA Mini Kit (Analytik Jena AG, Germany). mRNA concentrations were determined by DS-11 Spectrophotometer/Fluorometer (DeNovix, USA). Maxima H Minus First Strand cDNA Synthesis Kit (Thermo Fisher Scientific Inc., USA) was used to synthesize cDNAs in a standard reverse transcriptase reaction. The cDNA samples were then used as templates for real-time PCR analysis, which was performed on an qTOWER 2.0 Standard real-time PCR Thermal Cycler (Analytik Jena AG, Germany). Specific primer sequences for the *Rankl, Vdr*, and *Gapdh* (glyceraldehyde 3-phosphate dehydrogenase), that was used as a reference gene, were designed by Primer BLAST software: *Rankl*—forward 5′-CCAGCATCAAAATCCCAAGT-3′; reverse 5′-TGAAAGCCCCAAAGTACGTC-3′; product length—201 bp; *Vdr*—forward 5′-TCATCCCTACTGTGTCCCGT-3′; reverse 5′-TGAGTGCTCCTTGGTTCGTG-3′; product length—161 bp; *Gapdh*—forward 5′-TGAACGGGAAGCTCACTGG-3′; reverse 5′-TCCACCACCCTGTTGCTGTA-3′; product length—307 bp. Target genes were amplified for 50 cycles using Maxima SYBR Green/ROX qPCR Master Mix (Thermo Fisher Scientific, Inc., USA). Data were normalized to an internal housekeeping gene *Gapdh* and then calculated as the fold change relative to control using the ΔΔCt method.

### Isolation of Splenocytes, Peripheral Blood, and BM Mononuclear Cells

Spleens from rats were removed aseptically, and placed in 3 ml of media (RPMI 1640 with 10% fetal bovine serum) in a small petri dish. Using sterile tweezers, the spleen was placed on a sterile wire mesh screen and then carefully pushed through it with the plunger of a 10 ml syringe into the petri dish without transferring the capsule of the spleen in the media. Screen was rinsed with the 3 ml of media and the spleen mixture was gently pipetted, transferred into the tubes, and centrifuged at 500 *g* for 10 min at +4°C. Final pellets of splenocytes were diluted to 1 × 10^6^ cells/ml and stored on ice until fixation (19). Peripheral blood mononuclear cells were isolated using Histopaque-1083 (Sigma, USA) as described in the standard manufacturer’s protocol. Briefly, 5 ml of PBS without calcium and magnesium were added to 3 ml of whole blood, collected in heparin, and mixed. 3 ml of Histopaque-1083 were added to a centrifuge tube, then 8 ml of the blood–saline mixture were carefully layered on top. The mixtures were centrifuged at 400 *g* for 30 min, the upper layer was aspirated to within 0.5 cm of the opaque interface containing the mononuclear cells. The opaque interface was carefully transferred to a clean centrifuge tube and 10 ml of PBS were added, centrifuged at 250 *g* for 10 min. Supernatant was discarded. Lymphocyte pellets were resuspended with 5 ml of PBS and gently mixed, then centrifuged at 250 *g* for 10 min. After repeating the last steps twice the resulting pellets were diluted to 1 × 10^6^ cells/ml and stored on ice until fixation. BM mononuclear cells were obtained as follows: rat femurs were excised, dissected, and BM cells were harvested by repeated flushing of the femoral cavities with 1 ml syringe using PBS 10 times. Total marrow isolates were collected by centrifugation at 400 *g* for 1 min at +4°C and resuspended in PBS. The resultant cell suspension was filtered through successive 70- and 40-µm nylon cell strainers followed by 3 washes at 400 *g* for 5 min at +4°C. Subsequently, red blood cells (RBC) were lysed with RBC Lysis Buffer (155 mM NH_4_Cl, 12 mM NaHCO_3_, 0.1 mM EDTA, pH = 7.4) and the BM mononuclear cells were washed twice, centrifuged, and final pellet was resuspended in PBS (1 × 10^6^ cells/ml) and stored on ice until fixation.

### Cell Fixation and Permeabilization

Cell fixation was performed using 4% paraformaldehyde (Sigma, USA) for 10 min, then cells were rinsed with PBS for three times. If permeabilization was required (GR, pNF-κB p65 and VDR), 0.1% Triton X-100 was used for 10 min, followed by repeated rinse steps with PBS for three times. Nonspecific binding was blocked by incubation with PBS/1% bovine serum albumin for 45 min at room temperature.

### Flow Cytometry and Confocal Microscopy

RANK-, VDR-, and GR-positive BM cells were quantified using flow cytometry, RANK- and pNF-κB p65-positive cells were visualized by confocal microscopy. To detect the RANK, VDR, pNF-κB p65 and GR-positive cells (surface expression of RANK or cytoplasmic/nuclear localization of VDR pNF-κB p65 and GR), fixed splenocytes or peripheral blood/BM mononuclear cells were incubated with anti-RANK (1:150; Santa Cruz Biotechnology, USA), anti-VDR (1:100; Santa Cruz Biotechnology, USA), anti-pNF-κB p65 (1:250; Santa Cruz Biotechnology, USA) anti-GR (1:100; Santa Cruz Biotechnology, USA) for 60 min, then washed three times and incubated with specific DyLight 488-conjugated goat anti-rabbit IgG antibody or Alexa Fluor 568-conjugated goat anti-mouse IgG (H + L) antibody for 45 min in the dark box. The percentage of positive cells as well as the level of fluorescence was measured by an EPICS XLTM flow cytometer (Beckman Coulter, USA) using the excitation/emission wavelengths of 495/515 nm. The level of fluorescence was calculated as the mean fluorescent intensity of positive cells and data were expressed as folds of control. Background fluorescence was assessed by staining with control isotype-matched antibodies. All data were analyzed using FCS Express software. Immunoflourescence cell staining for confocal microscopy was performed as described above. Additionally, cell nuclei were visualized by Hoechst. Diode 405-30 laser (for Hoechst), Tunable Argon 458/477/488/514 nm at 30 mW laser (for DyLight 488) and He-Ne 543 nm at 1 mW (for Alexa Fluor 568) were used. Fluorescence was detected using the following channels: 420–480 nm, 505–530 nm, and >560 nm, respectively. Images were acquired using Carl Zeiss LSM 510 Meta confocal laser scanning microscope (Carl Zeiss, Germany) at 40× or 100× magnification and processed using Zeiss LSM Image Browser software. Laser power and the detector settings were kept constant to maintain consistency in the data collection system. For visualization studies, 10 slides were examined in random fields in at least three experiments. Based on the series of pictures obtained by scanning (with a step of 0.32 µm) of single RANK- or pNF-κB p65-positive cells (at least five in each group) the 3D models of RANKL/RANK interaction and pNF-κB p65 translocation into the nucleus were build using Zeiss LSM Image Browser software.

### ELISA Blood Serum 25-Hydroxyvitamin D Level Assay

Commercial ELISA kit “25-OH-Vitamin D_3_” (Immunodiagnostics, Germany) was used in accordance with the manufacturer’s instructions to assess the vitamin D_3_ status by determining of 25-hydroxyvitamin D (25OHD) concentration in rat serum.

### Statistical Analysis

Data distribution was analyzed using the Kolmogorov–Smirnov normality test. Normally distributed data are expressed as the mean ± SEM. Statistical differences between the groups were analyzed by the one-way ANOVA test. Differences were considered to be statistically significant when a *p*-value was less than 0.05. All statistical analysis was performed using Origin Pro 8.5 (OriginLab Corporation, Northampton, MA, USA).

## Results

### Assessment of the RANKL/RANK/OPG Axis and Quantifiation of Preosteoclasts in the BM

**Figure 1:**
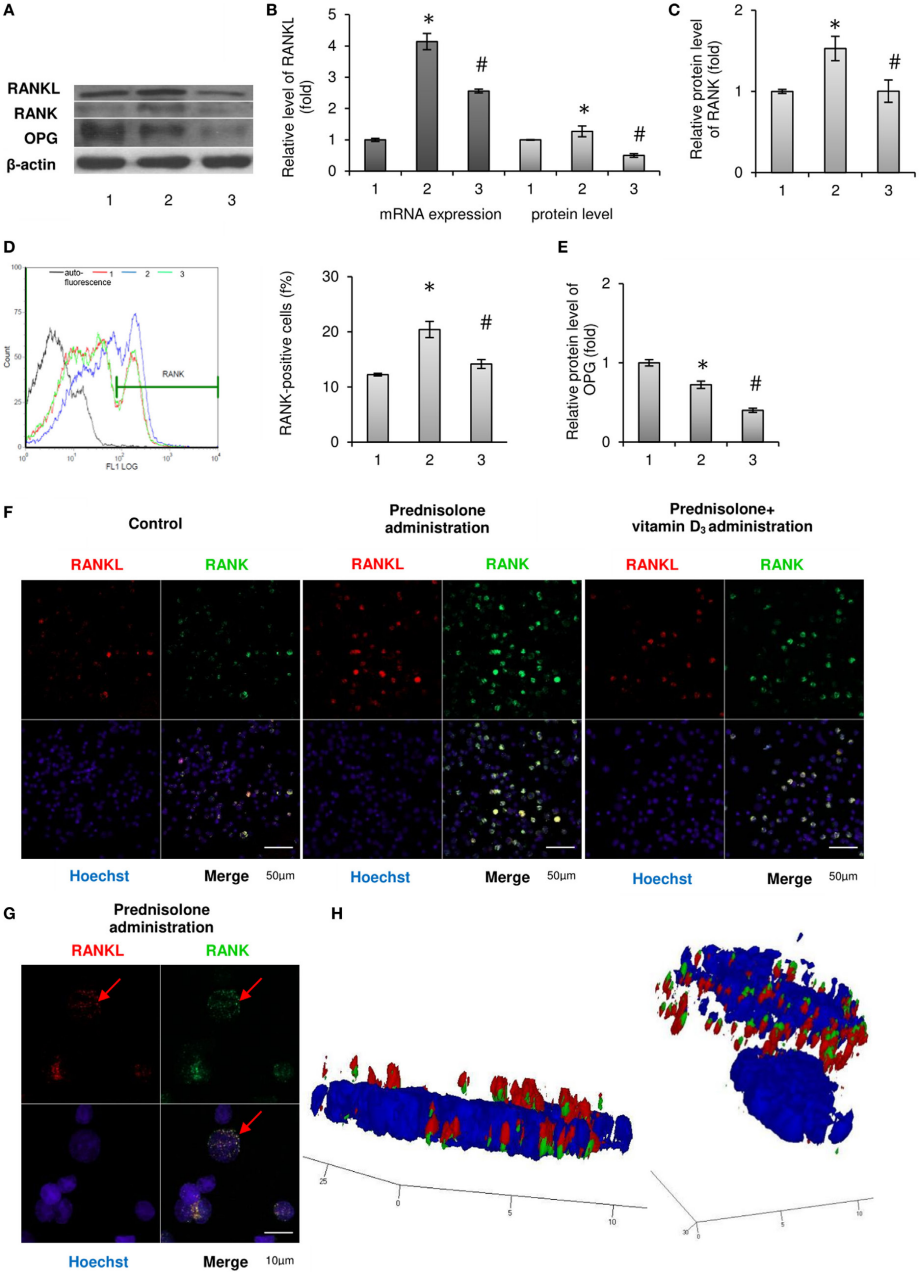
Effects of prednisolone and vitamin D_3_ administration on the RANKL/RANK/osteoprotegerin (OPG) signaling pathway: 1—control; 2—prednisolone administration (5 mg/kg of b.w.); and 3—prednisolone and vitamin D_3_ (1,000 IU/kg of b.w.) administration. Immunoblotting analysis of RANKL, RANK, and OPG in rat bone marrow (BM): representative immunoblots are shown **(A)** and quantified using β-actin as a loading control for total BM lysates. The bar graphs of RANKL **(B)**, RANK **(C)**, and OPG **(E)** are presented as means ± SEM (*n* = 6/group). Quantitative polymerase chain reaction of *Rankl* in rat BM **(B)**: data were normalized to *Gapdh* and pooled from two independent experiments (*n* = 6 rats/group). RANK-positive BM cells **(D)**: representative histograms (count—the number of events; FL1 LOG—fluorescence intensity) and quantification of RANK^-^positive cells documented by flow cytometry analysis. All data are shown as means ± SEM; *p < 0.05 vs. control, ^#^*p* < 0.05 vs. prednisolone administration. Immunocytochemical analysis of RANKL-positive (red fluorescence) and RANK-positive (green fluorescence) BM cells **(F)**. Hoechst (blue fluorescence) was used for nuclear staining. Scale bars indicate 50 µm (magnification 40×). Red arrows indicate co-localization of RANK and RANKL in BM osteoclast precursors in prednisolone group, suggesting RANKL–RANK direct interaction. Acquiring 3D model of RANKL–RANK interaction on the surface of BM cell in prednisolone-administered rats: based on the series of pictures obtained by scanning (with a step of 0.32 µm) of single RANK-positive cells (at least 5, scale bars indicate 10 µm, magnification 100×) **(G)** the 3D model of RANKL–RANK interaction **(H)** were build using Zeiss LSM Image Browser software.

The results of the study presented in Figures 1A,B show that a long-term prednisolone administration caused an increase in the *Rankl* mRNA and RANKL protein content in the BM by 4.14- and 1.27-fold (*p* = 0.0003 and *p* = 0.0007), respectively, compared with the control. At the same time, western blot analysis data revealed a 1.38-fold (*p* = 0.0042) lower level of OPG in the BM of prednisolone-administered rats than in control animals (Figures 1A,E). Physiological effects of OPG and RANKL on the skeletal system cells are known to be largely determined by the ratio of their synthesis (20). It was established a significant (2.0-fold) decrease in the OPG to RANKL proteins ratio induced by the prolonged action of prednisolone compared with the control. A decreased ratio of OPG/RANKL may contribute to the differentiation and activation of osteoclasts responsible for the enhancement of bone resorption. Vitamin D_3_ treatment caused a 1.6- and 2.4-fold decrease in the *Rankl* mRNA and RANKL protein levels (*p* = 0.0003 and *p* = 0.0007), respectively, as compared with prednisolone-administered rats. Although it has been found that cholecalciferol had a slight lowering effect (by 1.22-fold, *p* = 0.0042) on the level of OPG in the BM compared with the action of prednisolone, its ultimate impact on OPG/RANKL was, nevertheless, changed toward normalization.

Given that RANK is constitutively expressed in mature osteoclasts of the bone tissue, and its level in the BM also reflects the amount of immature precursors of osteoclasts, we examined the RANK protein expression, as well as the number of RANK-positive cells in the BM. It was shown that prednisolone upregulated (by 1.53-fold, *p* = 0.01) the relative RANK protein content in the BM, as compared with the control (Figure 1C). Consistent with the data obtained by flow cytometry, this effect was due to an increase in the number of osteoclasts precursors expressing RANK on their surface. Figure 1D illustrates that prednisolone induced a 1.7-fold (*p* = 0.0007) increase in the quantity of RANK-positive cells in the BM compared with the control. In addition, RANK-positive precursors of osteoclasts were visualized by confocal microscopy using indirect immunofluorescence labeling and the changes similar to those established by flow cytometry were confirmed (Figure 1F).

Since the effect of prolonged administration of GCs on the circulating pool of OCPs is not yet clarified, it was essential, apart from the BM, to estimate the number of RANK-positive cells among the monocytes/macrophages of the peripheral blood and spleen. Quantitative cytometric analysis of RANK-positive cells in the monocytes fraction isolated from rat peripheral blood and spleen showed an increase in their number by 4.2- and 1.26-fold (*p* = 0.049 and *p* = 0.012), respectively, as compared with the control (Table 1). Consequently, prednisolone induced more pronounced changes in the quantity of RANK-positive cells in the peripheral blood than in the spleen and BM.

**Table 1:** RANK-positive mononuclear cells from spleen, blood and bone marrow (BM).

Groups	Control	Prednisolone administration	Prednisolone ± vitamin D^3^ administration
RANK-positive cells isolated from spleen (%)	3.60 ± 0.12	4.53 ± 0.50*	2.36 ± 0.19^#^
RANK-positive mononuclear cells isolated from whole blood (%)	1.14 ± 0.05	4.08 ± 0.92*	1.87 ± 0.09^#^
RANK-positive mononuclear cells isolated from BM (%)	12.23 ± 0.24	20.42 ± 1.46*	14.17 ± 0.81^#^

Results represent the percentages of RANK-positive cells determined by flow cytometry. Values represent the means ± SEM for three experiments (M ± m, *n* = 7) done in triplicate. *p < 0.05 vs. control; ^#^*p* < 0.05 vs. prednisolone administration.

When vitamin D_3_ was co-administered with prednisolone, the relative content of RANK protein in the BM cell lysates was completely normalized, presumably due to the established decrease in the number of RANK-positive cells. Notably, the quantity of preosteoclasts in the BM reached the level that was found in control rats. The pool of circulating RANK-positive preosteoclasts after the administration of vitamin D_3_ almost returned to the control level in the peripheral blood, whereas in the spleen it reached values below the control.

Based on available scientific evidence that stromal cells are considered as one of the sources of RANKL, and RANK-positive cells are targets for regulatory control of this cytokine, we have used double (RANK plus RANKL) immunofluorescence labeling of the BM cells. Images obtained by confocal microscopy clearly demonstrate the co-localization of RANKL with the RANK-positive cells (Figure 1F). In addition, by scanning single cells with a step of 0.32 µm, a 3D model was constructed using the resulting series of images. One can notice the presence of RANKL bound to its receptor in RANK-positive cells isolated from animals that were given prednisolone (Figures 1G,H). It is also noteworthy that cells other than RANK-positive showed negligible immune-specific signal from RANKL.

Thus, prednisolone increased the number of circulating OCPs that was accompanied by the activation of the RANKL/RANK/OPG axis. Vitamin D_3_ significantly reduced the preosteoclastic pools and partially normalized the RANKL/RANK/OPG levels.

### NF-κB-Mediated Signaling in the BM

To address whether GC-induced excessive RANKL levels in the BM are implicated in upstream transcriptional activation through RANK signaling pathway, we explored the involvement of NF-κB in the mechanism of GC action and assessed potential therapeutic efficacy of vitamin D_3_.

**Figure 2:**
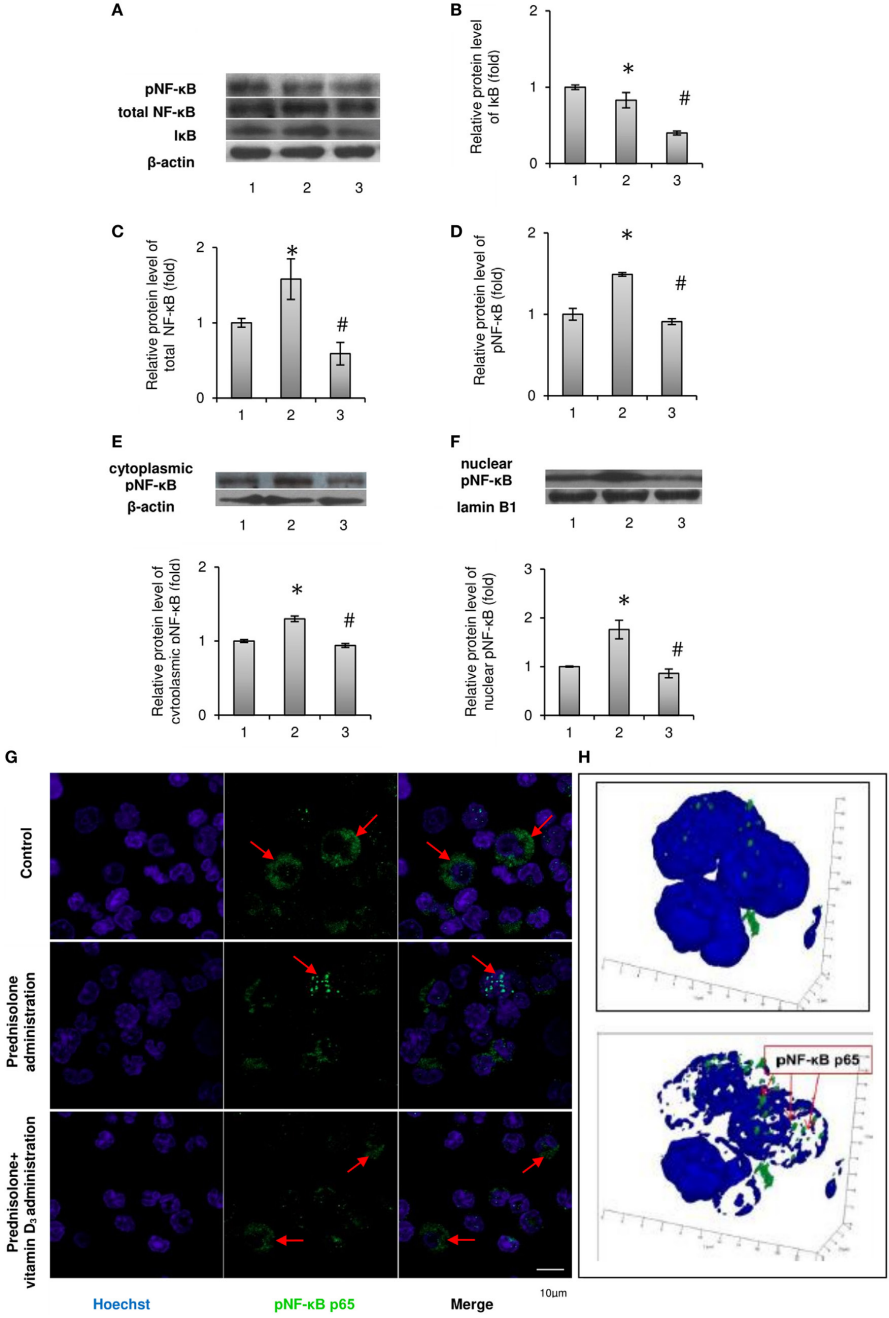
Levels of total, phosphorylated NF-κB p65 (Ser311) and IκB-α in rat bone marrow (BM): 1—control; 2—prednisolone administration (5 mg/kg of b.w.); and 3—prednisolone and vitamin D_3_ (1,000 IU/kg of b.w.) administration. Immunoblotting analysis of phosphorylated at Ser311, total NF-κB p65, and IκB-α in whole BM lysates: representative immunoblots are shown next to the bar charts **(A)** and quantified using β-actin as an internal control. The bar graphs of IκB-α **(B)**, total NF-κB p65 **(C)**, and phosphorylated at Ser311 **(D)** are presented as means ± SEM (*n* = 6/group). Immunoblotting analysis of NF-κB p65 subunit phosphorylated at Ser311 in cytoplasmic **(E)** and nuclear **(F)** lysates: representative immunoblots are shown above the bar charts and quantified using β-actin and lamin B1 as the loading controls for the cytoplasmic and nuclear fractions, respectively. The bar graphs of phosphoNF-κB p65 in cytoplasmic and nuclear lysates are presented as means ± SEM (*n* = 6/group),*p < 0.05 vs. control, ^#^*p* < 0.05 vs. prednisolone administration. Immunocytochemical analysis of phosphoNF-κB p65-positive (green fluorescence) BM cells **(G)**. Hoechst 33342 (blue fluorescence) was used for nuclear staining. Scale bars indicate 10 µm (magnification 100×). Red arrows show diffuse cytoplasmic distribution of phosphoNF-κB in the control and prednisolone + vitamin D_3_ administration groups and the nuclear localization of phosphoNF-κB in the prednisolone group. Acquiring 3D model of phosphoNF-κB nuclear translocation in BM cells in prednisolone-administered rats: based on the series of pictures obtained by scanning (with a step of 0.32 µm) of single phosphoNF-κB-positive cells (at least 5, magnification 100×) the 3D model of phosphoNF-κB nuclear translocation **(H)** were build using Zeiss LSM Image Browser software.

It is known that after the phosphorylation of main isoform of inhibitory nuclear factor κB (IκB-α) by the IκB kinase (IKK) complex, IκB-α is degraded and releases NF-κB, which translocates to the nucleus (21). Therefore, we first analyzed the effect of prednisolone on the IκB-α level in BM lysates and demonstrated its reduction by almost 1.2-fold (*p* = 0.0009) in GC-administered rats compared with control animals (Figures 2A,B). IκB-α level paradoxically further significantly decreased following the treatment with vitamin D_3_. The levels of total NF-κB p65 and pNF-κB p65 estimated in whole lysates of the BM cells were also found to be elevated by 1.58- and 1.48-fold (*p* = 0.047 and *p* = 0.0007), respectively, after prednisolone administration (Figures 2C,D). To determine the activation and nuclear translocation of NF-κB, the protein content of Ser311 phosphorylated large subunit of NF-κB (pNF-κB p65) was next quantified by western blot analysis in cytosol and nuclear fractions of the BM cells. Prednisolone elevated the levels pNF-κB p65 in cytoplasm and nuclei (Figures 2E,F) of the BM cells by 1.30- and 1.77-fold (*p* = 0.00001 and *p* = 0.00015), respectively, indicating a significant GC-elicited shift of pNF-κB p65 localization from the cytosol to the nuclear fraction of the cells. Nuclear translocation of pNF-κB is consistent with the probable transcriptional activation.

Vitamin D_3_ treatment diminished the levels of both phosphorylated and non-phosphorylated large subunits of NF-κB in whole lysates of the BM cells and partially blocked pNF-κB p65 translocation to the nucleus. The level of phosphorylated NF-κB p65 in the nuclear fraction was decreased to a greater extent than in cytosolic fraction, indicating a successful prevention of pNF-κB translocation and probable inhibition of its transcription activity.

In order to further confirm the translocation of pNF-κB p65 to the nucleus associated with prednisolone action, not using exclusively western blot analysis, the pNF-κB-positive BM cells were visualized by confocal microscopy after their indirect immunofluorescence labeling (Figure 2G). Corresponding to the results obtained in immunoblotting studies of pNF-κB p65, GC therapy significantly increased total immunofluorescence of pNF-κB p65 in the BM cells. Based on the scans of single pNF-κB-positive cells and the resulting series of images, a 3D model of pNF-κB translocation to the nucleus was constructed (Figure 2H). Our data confirmed the stimulatory effect of prednisolone on the translocation of pNF-κB to the nucleus, while the transcription factor remained more diffused in the cytoplasm from rats of the control and vitamin D_3_-administered groups.

In summary, we found GC-induced increase in the levels of both NF-κB phosphorylated and non-phosphorylated p65 subunits in BM cells and demonstrated that vitamin D_3_ administration attenuated transcriptional activation of the NF-κB p65.

### The BM Levels of GC Receptors

**Figure 3:**
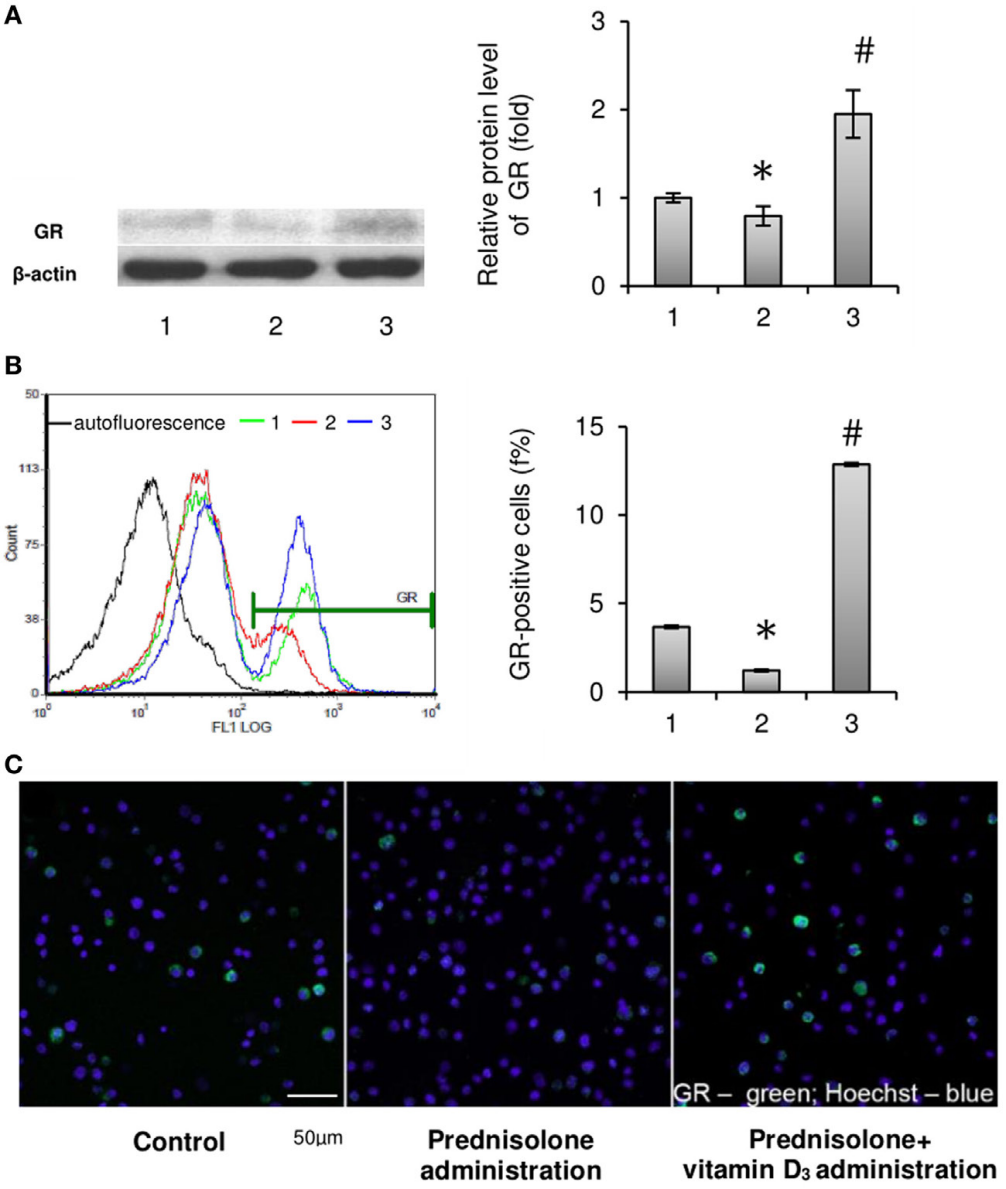
Effects of prednisolone and vitamin D_3_ administration on the level of glucocorticoid receptors in rat bone marrow (BM): 1—control; 2—prednisolone administration (5 mg/kg of b.w.); and 3—prednisolone and vitamin D_3_ (1,000 IU/kg of b.w.) administration. Proteins from total BM lysates were separated by SDS-PAGE and western blot analysis was performed using the antibodies against GR. Representative immunoblot and quantification of three experiments **(A)** are shown. Protein levels were normalized to β-actin. The bar graphs of GR are presented as means ± SEM (*n* = 6/group). GR-positive BM cells: representative histograms (count—the number of events; FL1 LOG—fluorescence intensity) and quantification of GR-positive cells documented by flow cytometry analysis **(B)**. All data are shown as means ± SEM; *p < 0.05 vs. control, ^#^*p* < 0.05 vs. prednisolone administration. Confocal microscopy of GR-positive (green fluorescence) BM cells **(C)** shows a significant decrease in the number of GR-positive cells after prednisolone administration. Hoechst 33342 (blue fluorescence) was used for nuclear staining. Scale bars indicate 50 µm (magnification 40×).

Considering that cell-specific effects of GCs are usually mediated by the GC receptor (22), it was advisable to determine the relative content of GRs in the BM. GC receptor protein levels evaluated in isolated BM cells showed a slight difference between GC-administered and control animals. Western blot analysis revealed a 1.26-fold (*p* = 0.0033) lowering effect of prednisolone on protein synthesis of GC receptors as compared with the control (Figure 3A). Consistent with the GC-induced downregulation of GR protein expression, there was a threefold (*p* = 0.00012) decrease in the number of GR-positive cells in the BM, which were quantified by immunofluorescence staining and flow cytometry and further visualized using confocal microscopy (Figures 3B,C). Low expression of GC receptors may indicate the involvement of the negative feedback mechanism of their regulation, or even desensitization, induced by excessive prednisolone load. Vitamin D_3_ supplementation had profound effect on the abundance of GRs and GR-positive cells. It increased GR protein and the amount of GR-positive cells to the levels 2.45- and 10.50 times (*p* = 0.0033 and *p* = 0.00012) as much as those in control rats. These results indicate that GR signaling in progenitor cells, playing a crucial role in coupling bone formation and resorption, could be effectively targeted by vitamin D_3_ co-administered with prednisolone.

### Characteristics of the Vitamin D Auto/Paracrine System in the BM

**Figure 4:**
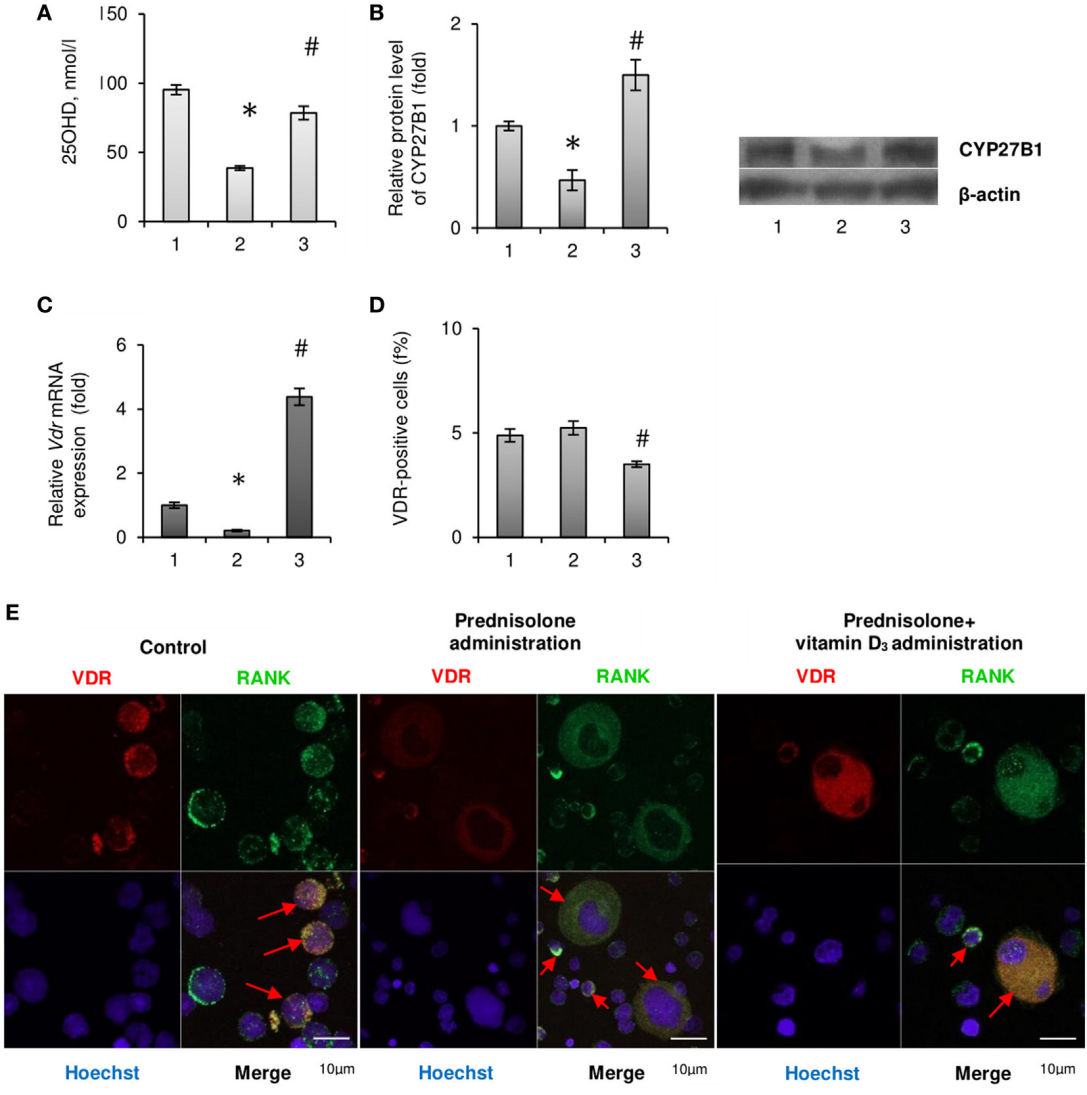
Vitamin D auto/paracrine system in bone marrow (BM) and vitamin D bioavailability after prednisolone and vitamin D_3_ administration: 1— control; 2—prednisolone administration (5 mg/kg of b.w.); and 3—prednisolone and vitamin D_3_ (1,000 IU/kg of b.w.) administration. 25OHD concentration was measured by ELISA **(A)**. Immunoblotting analysis of CYP27B1 in rat BM: the bar graphs of CYP27B1 **(B)** are presented as means ± SEM (*n* = 6/group) and representative immunoblots are shown next to the bar chart and quantified using β-actin as a loading control. Quantitative polymerase chain reaction of *Vdr* in rat BM **(C)**; data were normalized to *Gapdh* and pooled from two independent experiments (*n* = 6 rats/group). Quantification of VDR-positive BM cells using flow cytometry analysis **(D)**. Data are shown as means ± SEM; *p < 0.05 vs. control, ^#^*p* < 0.05 vs. prednisolone administration. Immunocytochemical analysis of double RANK-positive (green fluorescence) and VDR-positive (red fluorescence) BM cells **(E)**. Hoechst 33342 (blue fluorescence) was used for nuclear staining. Scale bars indicate 10 µm (magnification 100×). Red arrows show co-localisation of RANK and VDR in BM mononuclear osteoclast precursors (OCPs) in control group as well as in multinuclear OCPs in prednisolone and prednisolone + vitamin D_3_ group.

Since most of the BM disturbances associated with the chronic effect of prednisolone have been significantly attenuated by the administration of vitamin D_3_, we carried out an assessment of 25-hydroxyvitamin D (25OHD) level in the blood serum as a reliable marker of vitamin D bioavailability. A significant reduction of 25OHD was found in the blood serum of GC-administered animals (by 2.46-fold, to 38.78 ± 1.03 nmol/l) compared with the control (95,42 ± 1,36 nmol/l, *p* = 0.00015), indicative of a severe GC-induced vitamin D deficiency (Figure 4A). Vitamin D_3_ supplementation to GC-administered animals resulted in almost full normalization of 25OHD level.

Consistent with the evidence that 1α-hydroxylase (CYP27B1), which catalyzes 1,25(OH)_2_D synthesis in the kidney, is also expressed in other tissues, we assumed that 25OHD may be converted to 1,25(OH)_2_D locally in the BM, where it could exert autocrine/paracrine regulatory effects. As shown in Figure 4B prednisolone caused significant (2.14-fold, *p* = 0.0037) decrease in the protein expression of CYP27B1 in the BM compared with control animals. Vitamin D_3_ treatment restored CYP27B1 to the level that was even 1.5-fold higher than in the control (*p* = 0.0037).

As the biological effects of hormonally active form of vitamin D_3_ in different cell types are mediated through specific receptors to 1,25(OH)_2_D—VDR, we also examined the expression of *Vdr* gene in the BM cells. Following prednisolone administration, the level of *Vdr* mRNA in BM was shown to be diminished by 5.0-fold (*p* = 0.0027), indicating a possible decrease in cell responsiveness to vitamin D_3_ action (Figure 4C). It was found a strong increasing effect of vitamin D_3_ on *Vdr* mRNA expression that reached the value 4.38-fold higher than in the control.

In addition, we used immunofluorescence staining of the BM cells for flow cytometric analysis and confocal microscopy to detect the quantity of cells that express vitamin D receptor (VDR-positive cells). GC administration showed the number of VDR-positive cells similar to that observed in the control, whereas their amount decreased below the control level after vitamin D_3_ treatment (Figure 4D). Finally, confocal microscopy was used to study the precursors of osteoclasts with the double immunofluorescence staining for RANK and VDR (Figure 4E). What is the most interesting is that among the BM cells isolated from rats of prednisolone group, in addition to mononuclear cells, a large number of multinucleated and fused RANK-positive cells (preosteoclasts) were identified. Such cells were not seen in the control animals and were rarely found in the group of rats which received both prednisolone and vitamin D_3_ (Figure 4E). These data demonstrate vitamin D-mediated modulation of GC’s effects on rat bone progenitor cells and identify a role for VDR and RANK as mediators of this process.

Collectively, we established that prednisolone induced the vitamin D deficiency and lowered the levels of key components of the vitamin D auto/paracrine system in the BM, while cholecalciferol supplementation normalized, at least partially, these parameters.

## Discussion

A large body of evidence has demonstrated increased risk of secondary osteoporosis associated with a chronic GC treatment. The findings from clinical and experimental studies suggest that GC-induced loss of skeletal mass arises from changes in the numbers of bone cells, altering a balance between osteoblast-dependent bone formation and osteoclast-mediated bone resorption (23). Disturbances in the bone tissue caused by GCs may also be closely related to deleterious changes in the vitamin D endocrine system. Here, we found that molecular mechanisms of GC-induced bone loss involve aberrant interaction of GR and VDR signaling pathway and associated impairments of RANK/NF-κB axis in the BM that most likely results in abnormal osteoclastogenesis/osteosynthesis coupling. In addition to the well-recognized role of vitamin D and its receptor in mineral metabolism and bone formation, we confirmed the positive effects of cholecalciferol on GC-induced dysfunctions of the BM osteoprogenitors. In this investigation, we followed the prevention paradigm to counter pathogenic alterations elicited by chronic prednisolone therapy using concurrent vitamin D_3_ administration.

Glucocorticoids exert their numerous effects in cells largely through GR, which is a ligand-regulated transcription factor and the member of nuclear hormone receptor superfamily (22). Most of our findings can, at least in part, be explained by the alterations of GRs expression and impairment of the GR signaling. Prednisolone evoked a decrease in protein synthesis of GRs in the BM and a significant drop in the quantity of cells capable of expressing detectable amounts of GRs. It has been recently proposed that transrepression mediates most of the beneficial effects of GCs, whereas transactivation promotes most of their adverse effects (24). We can hypothesize that negative impact of chronic prednisolone therapy on cellular function may be mediated through insufficient interaction of GC receptors with prooxidant and/or inflammatory transcription factors to repress transcriptional activity, i.e., transrepression. Additionally, the underlying mechanism for prednisolone-associated abnormalities may also be attributable to the inhibitory action of the hormone on the expression of factors that limit the manifestation of side effects.

Osteogenesis is known to be a highly regulated process, in which subpopulations of BM cells differentiate into mature bone cells of skeletal tissues (25). Precursors of main bone-forming cells (osteocytes and osteoblasts) are mesenchymal stem cells residing in the BM. Osteoclasts originate from precursors of the myeloid/monocyte lineage and circulate in the monocyte fraction of the peripheral blood until they become resident cells of the bone tissue (26). GCs modify osteoclastic cell differentiation, number, and function either directly or through influencing the recruitment of their precursors from the BM, but the precise mechanisms are contradictory and not fully defined (27). RANK is known to be a key component of the osteokine system RANKL/RANK/OPG, localized on the cell surface of preosteoclasts, which facilitates their differentiation into osteoclasts and activation of mature osteoclasts responsible for bone resorption (20). Therefore, the RANK protein content strongly correlates with the number of osteoclastic cells and their activity in the bone tissue, and is also considered a reliable marker of preosteoclasts. With an augmented level of RANK protein currently established in the BM, we detected a significant increase in the number of RANK-positive cells (preosteoclasts) among the isolated BM cells. Moreover, prednisolone-induced migration of OCPs from the BM and the appearance of their significant amounts in the peripheral blood and spleen were also shown in our study. These results are in accordance with the suggestion that spleen can act as the reservoir of hematopoietic OCPs under pathological conditions (28). Although the mechanism underlying the massive recruitment of preosteoclasts into the peripheral blood and the redistribution of their pool between the BM, bloodstream, and spleen remains unclear, the findings indicate abnormal RANK-mediated regulation of the maturation of osteoclast BM progenitors following GC administration. In all probability, elevated number of RANK-positive cells in the BM indicates prednisolone ability to stimulate proliferation of osteoprogenitor cells, which is very likely associated with GR desensitization and decreased cellular signaling *via* GR.

We established the corrective effect of vitamin D_3_ on the pool of circulating RANK-positive precursors of osteoclasts, which is generally consistent with the antiproliferative activity inherent to this molecule (29). The effects of cholecalciferol may be due to its ability to relieve the arrest of the cell cycle of RANK-positive OCPs, the so-called “cell cycle-arrested quiescent osteoclast precursors” that can switch the differentiation of these cells from osteoclasts to dendritic cells (30).

The receptor activator of the NF-κB ligand is the central player in the regulation of osteoclastogenesis, and the quantity of RANKL presented to OCPs is essential for determining the intensity of osteoclast formation. The proper balance between the bone formation and resorption is reliably maintained by an adequate OPG to RANKL ratio. In the bone tissue OPG is synthesized mainly by osteoblasts and osteocytes and acts as an endogenous soluble decoy receptor for the RANKL (31). OPG, by binding RANKL, prevents RANK activation on the cell surface of the preosteoclasts and reduces both osteoclastogenesis and resorptive activity of mature osteoclasts. The harmonized effect of these cytokines was substantially disturbed by the administration of prednisolone, which exhibited a stimulatory effect on the formation of RANKL with a significant reduction in the content of OPG. Consequently, the detected reduction in the ratio of OPG/RANKL most likely contributes to the elevation of RANKL-mediated osteoclastogenesis.

Within the context of GC-induced deregulation of the RANKL/RANK/OPG axis, the possible relationship between BM progenitor cells and bone metabolism can be discussed. A number of research efforts unambiguously indicate that osteoblasts play a crucial role in the occurrence of secondary osteoporosis associated with chronic GCs treatment primarily due to hypofunction and increased apoptosis of osteoblasts and their progenitors (32). As for osteoclastogenesis, available scientific data indicate that both the decrease and increase in this process can account for the bone loss, provided that there is a concomitant decline in the formation and functional ability of osteoblasts (33, 34). Several recent studies which largely correspond to experimental design of the present investigation has demonstrated that chronic exposure of high GC doses had inhibiting effects both on bone formation and bone resorption (35, 36). The discrepancy between prednisolone-induced osteoclastogenic profiles of cytokines, the increase in the number of circulating RANK-positive cells, and the low bone turnover can be explained by suppressed recruitment of OCPs from the BM to the bones. In particular, GCs were reported to inhibit osteoclastogenesis through the downregulation of beta3 integrin, which plays an important role in the formation of multinucleated osteoclasts (37).

Vitamin D_3_ treatment was found to partially normalize altered RANKL level as well as the OPG/RANKL ratio that is consistent with the genomic or non-genomic VDR-modulated effects of the prohormone on cytokines production and cell-to-cell communication. The corrective effect of the compound points to vitamin D_3_ as a potential modulator of the pool of circulating OCPs both in the BM, from which they originate, and in the peripheral blood and spleen.

Because preosteoclastic cells are repoted to be a major target for RANKL, we also studied the regulatory mechanisms of RANKL subcellular signaling in isolated BM cells. The process of osteoclast formation requires the involvement of signaling *via* NF-κB activated in response to a key osteoclastogenic cytokine, RANKL, which controls the activation and maturation of osteoclasts through binding to RANK. RANKL and some proinflammatory cytokines, including TNFα, activate the NF-κB-associated signaling pathways, thereby positively regulating osteoclast formation and function (38).

Normally, the association of GR with the proinflammatory transcription factor NF-κB antagonizes its activity and is reported to be a primary mechanism by which GCs suppress inflammation (39). Several studies suggest that the GR and the p65/p50/inhibitory κB-α complex directly interact and GC/GR binding leads to the inhibiting of p65 transactivation function (40). However, the beneficial or deleterious role of GC/GR signaling in bone homeostasis seems to depend on the dose as well as treatment duration. Our findings convincingly indicate that chronic prednisolone administration induced transcriptional activation of the NF-κB signaling cascade in BM cells by the “classical” pathway, as is evidenced from increased total NF-κB p65 level and enhanced nuclear translocation of phosphorylated at serine 311(active) NF-κB p65 form. Also as expected, prednisolone destroyed NF-κB p65 sequestration complex with IκB-α, that promoted downstream NF-κB signaling. We can suggest that reduced protein level and possible resistance of GRs due to prolonged GC treatment may cause impaired interaction between the GR and p65/p50/IκB complex leading to the elimination of the inhibitory control of GR necessary to block NF-κB p65 transactivation.

The mechanism of osteoclasogenesis in prednisolone-induced osteoporosis can additionally be discussed considering NF-κB of BM cells as a target for regulation by the protein kinase Cξ (PKCξ) signaling. It has been recently reported that TNF-α-dependent NF-κB transcriptional activation may result from the PKCξ-mediated Ser311 phosphorylation of NF-κB p65 (41). As both RANKL and TNF-α belong to the same superfamily of cytokines, it is not excluded that RANKL may have similar effect on NF-κB Ser311 phosphorylation. It is possible that increased Ser311 phosphorylation of NF-κB p65 following prednisolone administration in our study, may occur in response to TNF-α stimulation and excessive reactive oxygen species formation.

We have shown that vitamin D_3_ can effectively inhibit prednisone-induced overactivation of the NF-κB signaling cascade in rat BM cells by modulating the NF-κB-associated signaling pathways and thereby promoting the normalization of osteoclastogenesis and restoring the balance between bone formation and resorption. Previously, Cohen-Lahav et al. (42) revealed that vitamin D_3_ can reduce NF-κB nuclear translocation by upregulating IκB-α level *via* increasing mRNA stability and decreasing IκB-α phosphorylation. In contrast to this report, our findings indicate that normalization of NF-κB signaling pathway was accompanied by a significant decrease in the protein level of IκB-α after vitamin D_3_ supplementation. Consistent with a marked vitamin D_3_-induced elevation of the GR level in the BM cells, we can speculate that cholecalciferol inhibits NF-κB activity by direct protein–protein GR/NF-κB interaction, preventing translocation p65 subunit to the nucleus (43). It restors the function of GR, which is recognized as a potent repressor of this transcriptional factor.

Vitamin D_3_ is one of the main regulators of bone tissue formation in the process of its development and remodeling throughout the life cycle. Through direct action on bone cells, the hormonally active form of vitamin D_3_ controls the proliferation of mesenchymal stem cells, their differentiation to osteoblasts and can directly or indirectly regulate the differentiation and activity of osteoclasts (17). Considering the presence of severe vitamin D_3_ deficiency following prolonged GC therapy and the effectiveness of cholecalciferol administration, the established changes in the BM may, at least in part, may be due to impairment of cholecalciferol biological effects in regulation of osteoclastogenesis and indicate direct or indirect VDR-mediated involvement of vitamin D hormone in these processes. According to our data, the negative effect of prednisolone includes abnormal vitamin D metabolism and impaired functioning of the vitamin D auto/paracrine system in the BM that can lead to deregulation of the VDR-mediated interaction of various cell types with the involvement of the RANKL/RANK/OPG cytokine system.

Importantly, synergistic anti-inflammatory and antiproliferative action of combined GC and cholecalciferol treatment has recently gained increased attention (44, 45). GCs have been previously shown to modulate effects of 1,25(OH)_2_D through regulation of VDR expression. Furthermore, numerous putative GC response elements were found in the *Vdr* gene (46). In whole, these facts contradict to adverse effects of prednisolone that have been successfully prevented by cholecalciferol in the present study. While GCs and 1,25(OH)_2_D may have similar effects on the cells, the specific mechanisms and pathways are probably involved, allowing vitamin D_3_ to counteract a harmful influence of prednisolone on rat BM. Vitamin D_3_ could act through separate mechanisms from those utilized by GCs, most likely through differences in transcriptional targets and through different effects on shared targets, providing benefits in treating prednisolone-induced BM disorders. Determining whether these shared and non-shared pathways, suggestive of potential mechanisms, mediate a combined effect of prednisolone and vitamin D_3_ will require further investigation.

## Conclusion

Prednisolone administration caused impairments of the VDR and GR signaling that contributed to the RANK/NF-κB axis activation in bone marrow cells and increased pool of circulating preosteoclasts. Sufficient vitamin D bioavailability and proper VDR expression are important for restoration of crosstalk between osteoclastogenic signaling pathways. Although clinical use of the study is restricted, novel development that have risen from our research in vitamin D biology and discoveries in the bone remodeling process can be expected to result in further treatment options based on vitamin D for various bone disorders.

## Data Availability Statement

Datasets are available on request: the raw data supporting the conclusions of this manuscript will be made available by the authors, without undue reservation, to any qualified researcher.

## Ethics Statement

All experiments for the research work “Vitamin D_3_ modulates impaired crosstalk between RANK and glucocorticoid receptor signaling in bone marrow cells after chronic prednisolone administration” were conducted in accordance with international recommendations of the European Convention for the Protection of Vertebrate Animals used for Research and Scientific Purposes (Strasbourg, 1986), General Ethical Principles of Animal Experimentation, approved by the First National Congress on Bioethics (Kyiv, 2001) and are ethically acceptable.

## Author Contributions

OL and IS contributed to the study design. OL, IS, AM, and DL collected and analyzed data. OL, IS, and MV interpreted the results and wrote and revised the final draft of the manuscript.

## Conflict of Interest Statement

All authors declare that the research was conducted in the absence of any commercial or financial relationships that could be construed as a potential conflict of interest.
